# BNT162b2 and ChAdOx1 nCoV-19 vaccinations, incidence of SARS-CoV-2 infections and COVID-19 hospitalisations in Scotland in the Delta era

**DOI:** 10.7189/jogh.12.05008

**Published:** 2022-03-26

**Authors:** Syed Ahmar Shah, Chris Robertson, Igor Rudan, Josephine LK Murray, Colin McCowan, Zoe Grange, Audrey Buelo, Christopher Sullivan, Colin R Simpson, Lewis D Ritchie, Aziz Sheikh

**Affiliations:** 1 Usher Institute, University of Edinburgh, Edinburgh, UK; 2 University of Strathclyde, Glasgow, Edinburgh, UK; 3 Public Health Scotland, Glasgow, Edinburgh, UK; 4 School of Medicine, University of St Andrews, St Andrews, UK; 5 School of Health, Wellington Faculty of Health, Victoria University of Wellington, Wellington, New Zealand; 6 Academic Primary Care, University of Aberdeen, Aberdeen, UK

## Abstract

**Background:**

The emergence of the B.1.617.2 Delta variant of concern was associated with increasing numbers of severe acute respiratory syndrome coronavirus 2 (SARS-CoV-2) infections and COVID-19 hospital admissions. We aim to study national population level SARS-CoV-2 infections and COVID-19 associated hospitalisations by vaccination status to provide insight into the association of vaccination on temporal trends during the time in which the SARS-CoV-2 Delta variant became dominant in Scotland.

**Methods:**

We used the Scotland-wide Early Pandemic Evaluation and Enhanced Surveillance (EAVE II) platform, covering the period when Delta was pervasive (May 01 to October 23, 2021). We performed a cohort analysis of every vaccine-eligible individual aged 20 or over from across Scotland. We determined the vaccination coverage, SARS-CoV-2 incidence rate and COVID-19 associated hospitalisations incidence rate. We then stratified those rates by age group, vaccination status (defined as “unvaccinated”, “partially vaccinated” (1 dose), or “fully vaccinated” (2 doses)), vaccine type (BNT162b2 or ChAdOx1 nCoV-19), and coexisting conditions known to be associated with severe COVID-19 outcomes.

**Results:**

During the follow-up of 4 183 022 individuals, there were 407 405 SARS-CoV-2 positive cases with 10 441 (2.6%) associated with a hospital admission. Those vaccinated with two doses (defined as fully vaccinated in the current study) of either vaccine had lower incidence rates of SARS-CoV-2 infections and much lower incidence rates of COVID-19 associated hospitalisations than those unvaccinated in the Delta era in Scotland. Younger age groups were substantially more likely to get infected. In contrast, older age groups were much more likely to be hospitalised. The incidence rates stratified by coexisting conditions were broadly comparable with the overall age group patterns.

**Conclusions:**

This study suggests that national population level vaccination was associated with a reduction in SARS-CoV-2 infections and COVID-19 associated hospitalisation in Scotland throughout the Delta era.

There have been reported severe acute respiratory syndrome coronavirus 2 (SARS-CoV-2) infections, and COVID-19 hospital admissions amongst double-vaccinated people since the emergence of B.1.617.2 delta variant of concern in countries with high vaccination coverage [1,2]. Even without the emergence of new variants, “breakthrough” infections, severe cases, and even fatalities among double vaccinated were not unexpected events among epidemiologists and within public health organisations since the randomised clinical trials of the vaccines did not demonstrate 100% efficacy [3,4]. It is, therefore, important to continue to gather accurate information in real time and analyse the emerging health data to help inform policy and the public about the pandemic.

In addition to vaccine effectiveness studies [1,5,6], reporting and interpreting national population level trends of SARS-CoV-2 infections and COVID-19 hospitalisations by vaccination status is also important as it helps us to understand the impact of the pandemic on the NHS and informs prioritisation of the rollout of the vaccination programme. When looking at this trend data, it is important to use appropriate denominators to calculate rates rather than raw case numbers which may be misinterpreted by the public and decision makers. To be able to investigate country-wide national trends of infection and COVID-19 associated hospitalisation requires linkage of multiple sources of quality health information in near real-time that can be accessed and analysed. Scotland is a good example where this is possible [1,7-10].

The aim of this study is to describe the COVID-19 vaccination roll out and the temporal trends in SARS-CoV-2 infection and COVID-19 associated hospitalisations in Scotland. We hypothesise that the incidence rate of SARS-CoV-2 infection and COVID-19 associated hospitalisation amongst vaccinated individuals will be substantially lower than incidence rates for individuals who are unvaccinated throughout the period when the Delta variant was dominant. However, these comparisons of COVID-19 case rates in those who are vaccinated and unvaccinated should not be used to assess how effective the vaccine is in preventing serious health outcomes. Formal vaccine effectiveness studies should be used for that, which aim to take account of confounding.

## METHODS

### Data source

We used the Scotland-wide Early Pandemic Evaluation and Enhanced Surveillance (EAVE II) platform, covering the period when Delta was pervasive [4]. The EAVE II platform has been used to investigate the effectiveness [1,5,6] and safety of COVID-19 vaccines [11,12], and inform public health policy deliberations in Scotland [7-10].

This platform makes use of the National Health Service mandated unique identifier (Community Health Index, commonly referred to as the CHI number) for every person to deterministically link multiple data sets in near real-time. These include data on COVID-19 vaccinations, reverse transcriptase polymerase chain reaction (RT-PCR) testing, serology, hospitalisations, and deaths. EAVE II contains data from 5.4 million people from across Scotland, providing around 99% coverage of the entire country’s population.

### Study design, data source and population

We undertook a retrospective cohort analysis with a follow-up from May 1, 2021, to October 23, 2021. All individuals who were deemed eligible for vaccination and aged 20 years or older on March 01, 2021, were included and followed up until death, or until the end of the study period.

### Data analysis

#### Exposure

**Figure 1:**
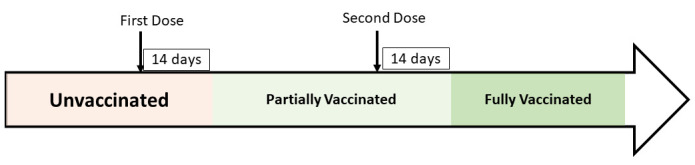
Determination of vaccination status: A person is considered unvaccinated until 14 days after having received first dose. Subsequently, the person is considered partially vaccinated until 14 days after receiving second dose.

The ‘independent’ variables of interest in the study were vaccine type (BNT162b2 or ChAdOx1 nCoV-19) and vaccination status. **Figure 1** graphically illustrates how we determined each person’s vaccination status. A person was assigned a vaccine status of “unvaccinated” until 14 days after having received their first dose. Subsequently, the person was assigned a vaccine status of “partially vaccinated” until 14 days after having received their second dose. Then, a person was assigned a vaccine status of “fully vaccinated”.

#### Outcome

**Figure 2:**
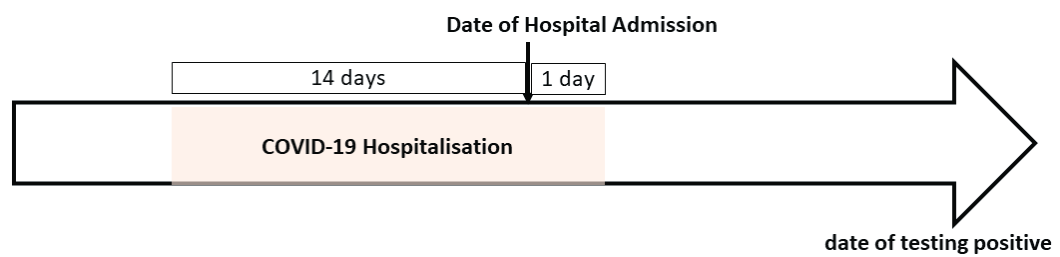
Determination of COVID-19 hospitalisation: For a given hospital admission, getting a SARS-CoV-2 positive test in the shaded time window (14 days before the admission, or on the day of admission, or on a day after admission) is considered to be associated with COVID-19 hospitalisation.

The two outcomes of interest in the study were confirmed infection with SARS-CoV-2 and COVID-19 hospitalisation. We used the RT-PCR test result to identify individuals who tested positive (includes both symptomatic and asymptomatic cases). We assessed each individual positive RT-PCR test result to determine if there was an associated hospitalisation. **Figure 2** graphically illustrates how we determined whether a given SARS-CoV-2 positive case was deemed to have led to hospitalisation. If a person was admitted to a hospital within 14 days after testing positive, or testing positive on the day of admission, or a day after admission, then this was considered a “COVID-19 hospitalisation”. With the chosen definition, we are able to capture ‘community-acquired’ infection while ensuring that potential nosocomial infections acquired during a hospital stay are not counted. We do not attempt to determine whether the hospitalisation was in relation to their COVID-19 infection or incidental to it.

Using the vaccination data, we are able to evaluate the cumulative numbers of individuals in each vaccination group at the beginning of each week during the study. From the linked vaccination, testing and hospitalisation data we can enumerate the numbers of individuals testing positive or admitted to hospital following a positive test, by vaccination status, for each week in the study. Effectively, our approach allows us to dynamically update all the numbers for each week during the follow-up. We estimated the incidence rate (IR) in each stratum, for every week, during the follow-up period. This means that a given IR estimate represents the total number of individuals who had an outcome (SARS-CoV-2 infection or COVID-19 associated hospitalisation) in a week divided by the total number of people at risk in each stratum considered.

#### Confounders

Two key confounders adjusted for were age and having other co-existing conditions (Q COVID risk groups [13,14]), both of which have previously been identified to increase the risk of COVID-19 hospitalisation or death. For age, we stratified the cohort population in four age groups (20-39, 40-59, 60-79, 80+). For the presence of co-existing conditions, we assigned each individual to one of the three risk categories depending on the number of recorded coexisting Q-COVID conditions that the individual had in their primary care records (0: “no recorded co-existing condition”; 1: “only one co-existing condition”; 2+: “at least two co-existing conditions”).

#### Total population estimation

In this study, we computed the total number of people in each stratum for every day. As an example, this meant that any individual only contributed to the total count until their death, and an individual only contributed to the total count of ‘unvaccinated’ group until the day of having a ‘partially vaccinated’ status. We computed both the daily and weekly IRs. We have preferred reporting the weekly IRs to better illustrate the overall population-level trends. The denominator to use when computing the weekly IR was the total number of individuals at the beginning of the respective week.

The GP population generally exceeds a mid-year population for a country, because some individuals are recorded in two general practices, whereas others are temporary visitors or may have left the country [15]. To ensure that any potential rogue records (that could arise due to the afore-mentioned factors among other reasons) are appropriately handled and do not lead to erroneous results, each individual record in the EAVE database was assigned a weight ranging from 0 to 1 that was then readjusted dependent on recent health care system interaction. This algorithm worked as follows:

- Assign a weight (≤1) to each individual in the database such that the summation of weights across the entire database is equal to the total, mid-year population estimate in Scotland by single year of age and gender [16]
- Identify all individual records who have any recent interaction with the healthcare system.
- For all individuals identified in (ii), assign them a weight of 1, and correspondingly, down-weight the rest such that the summation of weights across the entire database is still equal to the known, total population in Scotland.
- Remove all patients who have been identified by Public Health Scotland as not eligible for vaccination – as they are no longer in the country.

All analysis was undertaken in RStudio server (version 1.1.463) using R (version 3.6.1). The tidyverse packages were used for data wrangling (dplyr), and plotting (ggplot2) [17].

### Ethics and permissions

The ethical approval for this study was granted by the National Research Ethics Service Committee, Southeast Scotland (reference number: 12/SS/0201). The Public Benefit and Privacy Panel for Health and Social Care approved the data linkage (reference number: 1920-0279) and hence individual, written consent was not required.

## RESULTS

This cohort analysis consists of 4 183 022 individuals (20-39: 1 410 800; 40-59: 1 469 937; 60-79: 1 079 080; 80+: 223 205) on May 01, 2021 who were then followed up until the end of the study (October 23, 2021) or until death if it was sooner. During this period, there were 407 405 individual SARS-CoV-2 positive cases (63.6% of all individual positive cases reported during the entire pandemic in Scotland until October 23, 2021). Out of the 407 405 positive cases, 10 441 (2.6%) were associated with hospitalisation.

**Figure 3:**
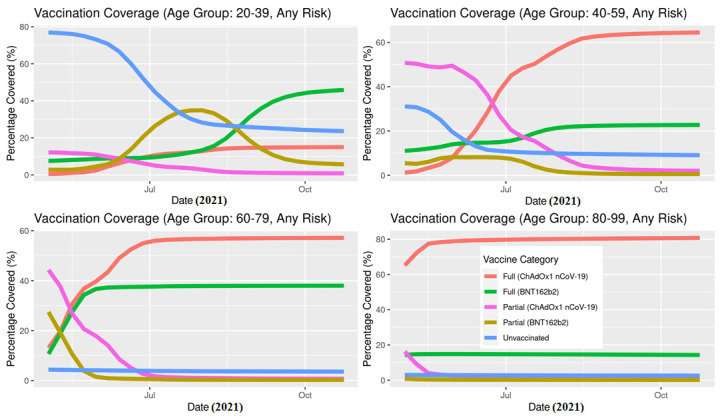
Vaccination coverage over time stratified by age, vaccination status and type in Scotland, May 12021 to October 23, 2021.

**Figure 4:**
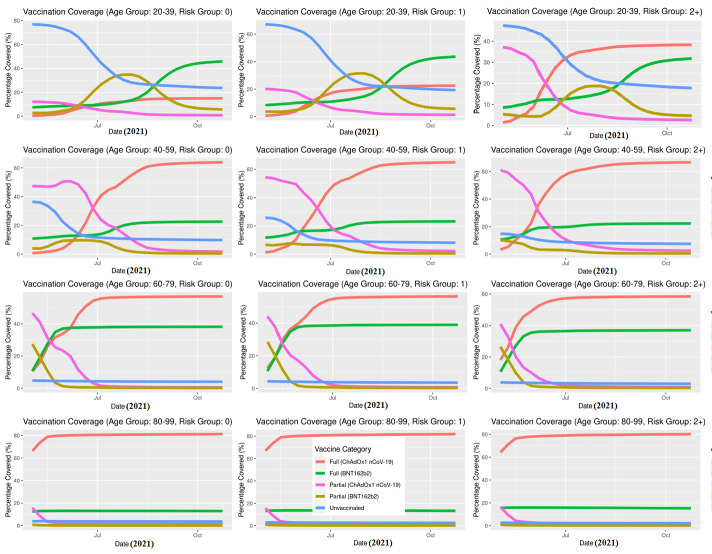
Vaccination coverage over time, further stratified by risk group in Scotland, May 1, 2021, to October 23, 2021.

At the start of the study period, 80.0% of 80+ year-olds were fully vaccinated (most received ChAdOx1 nCoV-19), 56.7% of 40-59 and 71.8% of 60-79 year-olds were partially vaccinated, and most 20-39 year-olds were unvaccinated (76.9%). At the end of the study period, the majority in all age groups were fully vaccinated (20-39: 68.6%; 40-59: 88.3%; 60-79: 95.5%; 80+: 95.7%) **(****Table 1****)**. The majority of 60-79 were fully vaccinated by July having received either ChAdOx1 nCoV-19 or BNT162b2, and most 40-59 were fully vaccinated by August with most receiving ChAdOx1 nCoV-19 (**Figure 3**). Individuals with 2 or more risk conditions (2+) were vaccinated earlier compared to those with 0 or 1 risk conditions, and the majority of these high risk individuals received ChAdOx1 nCoV-19 across all age groups (**Figure 4**).

**Table 1:** Percentage of unvaccinated, partially vaccinated and fully vaccinated people on May 1, 2021, (study start date) and October 23, 2021 (study end date)

	May 01, 2021			October 23, 2021		
	**UV (%)**	**PV (%)**	**FV (%)**	**UV (%)**	**PV (%)**	**FV (%)**
Age (20-39)						
all	76.9	15.1	8.0	23.6	7.8	68.6
risk group 0	82.0	10.4	7.5	25.4	7.7	66.9
risk group 1	67.2	23.8	9.0	19.2	8.1	72.7
risk group 2+	47.5	42.7	9.8	17.7	8.2	74.1
Age (40-59):						
all	31.1	56.7	12.2	9.1	2.6	88.3
risk group 0	36.4	52	11.6	9.8	2.4	87.8
risk group 1	25.8	61.3	13	8.1	2.9	89
risk group 2+	14.8	71.3	13.9	7.4	3.1	89.4
Age (60-79):						
all	4.4	71.8	23.8	3.6	1	95.5
risk group 0	4.8	74	21.3	4	0.8	95.2
risk group 1	4.3	72.7	23	3.5	1	95.6
risk group 2+	3.8	67.5	28.7	3	1.2	95.8
Age (80+):						
all	3	17	80	2.5	1.8	95.7
risk group 0	4	16.7	79.3	3.6	1.6	94.8
risk group 1	2.9	16.6	80.5	2.4	1.9	95.7
risk group 2+	2.6	17.3	8	2.2	1.8	96

UV – unvaccinated, PV – partially vaccinated, FV – fully vaccinated

**Figure 5:**
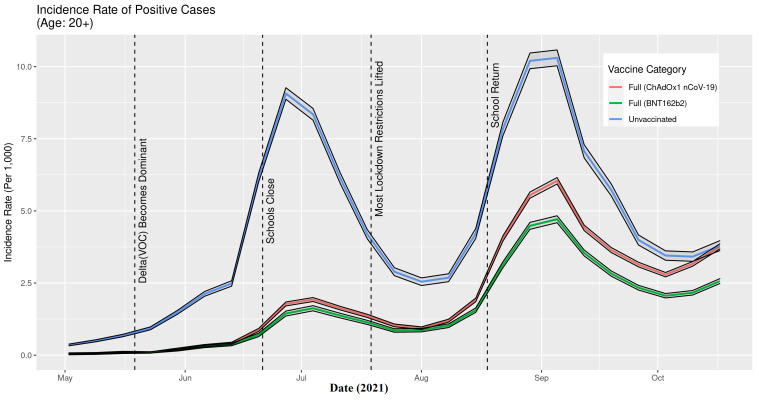
Incidence rate of SARS CoV-2 infections in Scotland in relation to key events marked by dashed vertical lines, May 1, 2021, to October 23, 2021. The 95% confidence intervals, for each time-series, are shown by the solid black lines.

**Figure 6:**
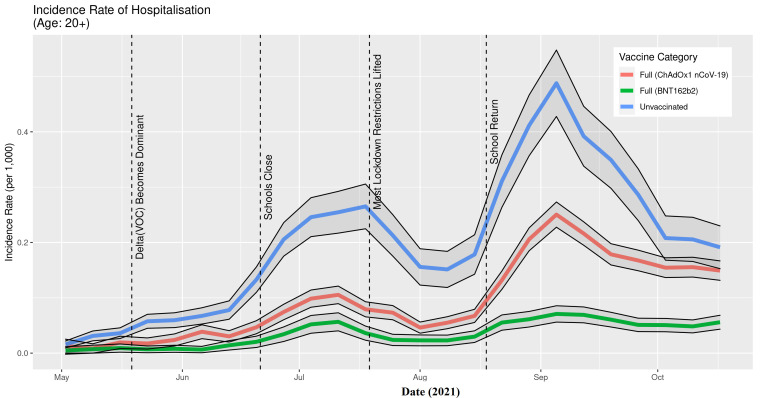
Incidence rate of COVID-19 hospitalisations in Scotland in relation to key events marked by dashed vertical lines, May 1, 2021, to October 23, 2021. The 95% confidence intervals, for each time-series, are shown by the solid black lines.

The IR of SARS-CoV-2 infections (**Figure 5**) and COVID-19 associated hospitalisation (**Figure 6**) during the period from mid-May – end of September, 2021, clearly and consistently show that the incidence rates of those fully vaccinated with either vaccine were lower than for those who were unvaccinated in the Delta era in Scotland. There is a lack of separation in October for infections and hospitalisations where the confidence intervals for unvaccinated and being fully vaccinated with ChAdOx1 nCoV-19 overlap and in early May for hospitalisations when all three lines overlap.

There were two distinct peaks observed during the study period: July and September. During the July peak, the IRs (total weekly count per 1000 people) of SARS-CoV-2 infections were 9.1 (unvaccinated), 1.9 (fully vaccinated with ChAdOx1 nCoV-19), and 1.6 (fully vaccinated with BNT162b2). The IR of COVID-19 hospitalisation during the same peak were 0.3 (unvaccinated), 0.1 (fully vaccinated with ChAdOx1 nCoV-19), and 0.06 (fully vaccinated with BNT162b2). During the September peak, the IRs of SARS-CoV-2 infections were 10.3 (unvaccinated), 6.0 (fully vaccinated with ChAdOx1 nCoV-19), and 4.7 (fully vaccinated with BNT162b2). The IRs of COVID-19 hospitalisation during the same peak were 0.5 (unvaccinated), 0.3 (fully vaccinated with ChAdOx1 nCoV-19), and 0.07 (fully vaccinated with BNT162b2).

**Figure 7:**
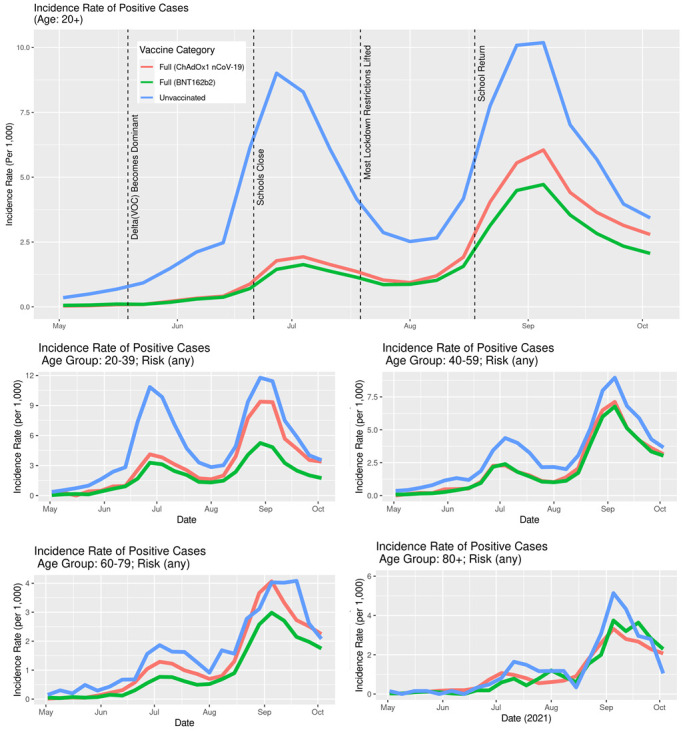
Incidence rates of SARS-CoV-2 positive cases in Scotland stratified by age, vaccination status and type, May 1, 2021, to October 23, 2021.

**Figure 8:**
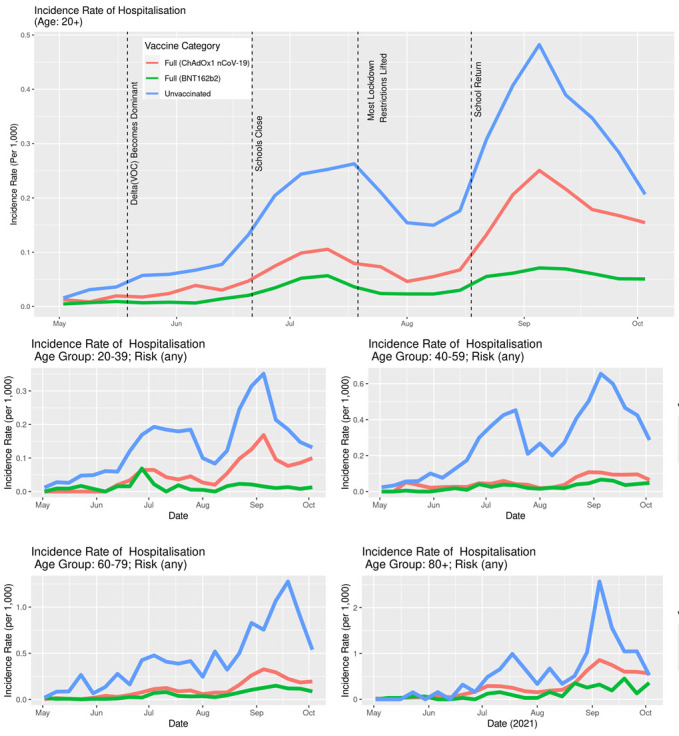
Incidence rates of COVID-19 hospitalisations in Scotland stratified by age, vaccination status and type, May 1, 2021, to October 23, 2021.

The IRs of SARS-CoV-2 infection stratified by age (**Figure 7**) show that the overall difference between vaccinated and unvaccinated was largely driven by the younger age groups where the incidence rates are higher. In the age group 60-79, the differences were less pronounced, while in the age group 80+, there was no notable difference. In both older age groups, a very small percentage of the population was unvaccinated (**Figure 3**). Unlike the IR for infection, the IR for hospitalisation stratified by age (**Figure 8**) clearly show that the older age group have a higher incidence rate.

Although there is considerable stochastic variation in such detailed analyses with increasing stratification (where number of individuals contributing to a given stratum in a time-series become increasingly smaller), the overall message is preserved: typically, the sub-population of unvaccinated people were associated with a greater risk of infection and hospitalisation than either of the two sub-populations of vaccinated individuals.

## DISCUSSION

**Figure 9:**
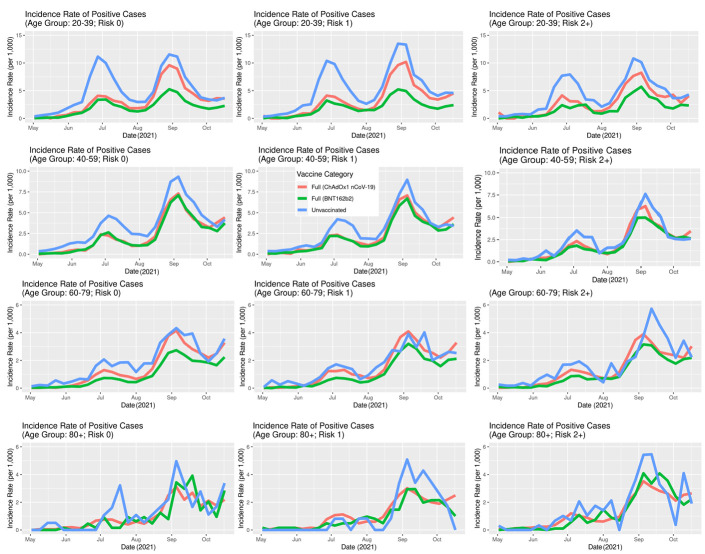
Incidence rates of SARS-CoV-2 positive cases in Scotland, further stratified by risk, May 1, 2021, to October 23, 2021.

**Figure 10:**
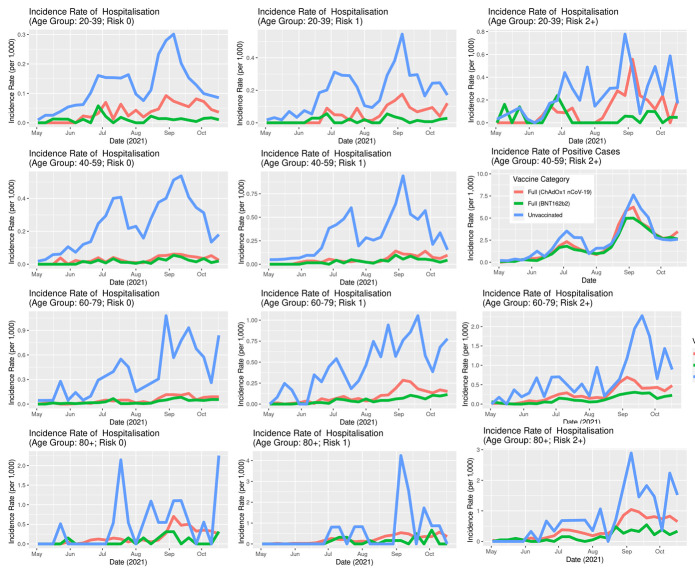
Incidence rates of COVID-19 hospitalisations in Scotland, further stratified by risk, May 1, 2021, to October 23, 2021.

Both the BNT162b2 and ChAdOx1 nCoV-19 offered protection against SARS-CoV-2 infection, and COVID-19 hospitalisation in Scotland throughout the Delta period. Overall, the IRs were consistently higher for the unvaccinated compared to those fully vaccinated with either vaccine (**Figures 5,6**). Previous studies have suggested both age and having other co-existing conditions as key risk factors for worse COVID-19 outcomes [13,14]. We consequently stratified our analyses by age and presence of other co-existing conditions. The stratified analyses (**Figures 7,8**) show that the risks of SARS-CoV-2 events, especially hospitalisation, are lower in the double vaccinated groups throughout the Delta era. These results were broadly comparable when we further stratified the different age groups by risk groups (**Figure 9** and **Figure 10**).

Our findings suggest that younger people were substantially more likely to get infected, especially if unvaccinated, compared to older age groups (**Figure 7**). Unlike the risk of getting infected, the risk of a severe COVID-19 outcome such as hospitalisation is substantially higher in older age groups. This likely explains the substantially higher IR of hospitalisations amongst the older age groups compared to the IR amongst the younger age groups (**Figure 8**).

The data presented here cannot be used to suggest that BNT162b2 offers better protection compared to ChAdOx1 nCoV-19, particularly for COVID-19 hospitalisation (**Figure 6**). These data need to be interpreted with caution and there are two reasons for this. To begin, the majority of adults over age 80 received ChAdOx1 nCoV-19, and they are at higher risk for severe COVID-19 outcomes. Second, these individuals received the ChAdOx1 nCoV-19 dose earlier in the vaccination roll-out than less vulnerable individuals (see **Figures 3 and 4**) and their vaccine-induced immunity may have waned [18], which is not accounted for in the current descriptive analysis. However, further analyses would be necessary to investigate the differential protection between BNT162b2 and ChAdOx1 nCoV-19 vaccines as the beginning of the vaccination programme is out with the study period of the present paper.

The key strength of our study lies in showing how access to multiple, country-wide health care data sets that were deterministically linked in near real-time permits the presentation of visually striking information on the patterns of vaccination uptake and SARS-CoV-2 infections and hospitalisation by vaccine status. Using data for a whole country yields a large sample size (over 4 million individuals), and hence permits adjustment for key confounders (age and number of co-existing conditions) using individual-level data. Another strength of our study is the ability to dynamically update the vaccination status of every individual and then use denominators to estimate incidence rates by vaccination type and status in near real-time.

However, there are some limitations to note. We have used the “Rapid Preliminary Inpatient Data” (RAPID) to identify COVID-19 hospitalisations. The RAPID data set was originally developed for monitoring and prediction of emergency admissions and bed occupancy in Scotland and does not contain detailed analyses of admission causes. In our analysis, therefore, we cannot differentiate between hospital admissions due to COVID-19 and with COVID-19 (situations where an individual was in the hospital for other reasons, but COVID-19 was incidentally identified). In our analysis though, and with our chosen definition of COVID-19 hospitalisation, any potential nosocomial infection acquired during a hospital stay is not counted.

Further, the number of cases that we have captured using the national-level surveillance data are affected by the testing strategy. Asymptomatic SARS-CoV-2 cases who do not get tested will not contribute to the total number of cases and the IRs computed in this study are therefore an approximation of the actual IR at any given time point. Further, our analysis did not take any post-infection immunity into account. In addition, while we have stratified our analyses by age and risk group, additional factors such as behaviour and testing may introduce systematic differences between vaccinated and unvaccinated populations. It is therefore important to interpret these results with caution and we have consequently chosen not to compute vaccine effectiveness estimates. Further, we defined a person to be fully vaccinated if they received their second dose. However, as the pandemic has now further prolonged, the vaccination roll-out has recently been extended to children, and booster doses are now offered to all adults [19]. We have not analysed the impact of any booster dose and the cohort has been restricted to all individuals of 20 years or older. Lastly, there is no consensus on the number of days chosen as cut-off before a person is deemed to have changed their vaccination status after receiving a dose. This is especially true for the cut-off of 14 days chosen after first dose when a person’s vaccination status is deemed to have changed from unvaccinated to partially vaccinated. As we have primarily focussed on comparison of temporal trends between unvaccinated and fully vaccinated thereby allowing several weeks of gap between unvaccinated and fully vaccinated status (see **Figure 1**), we believe that the cut-off chosen for when a person’s status switches from unvaccinated to partially vaccinated will not change the overall conclusions in this study.

## CONCLUSIONS

In summary, these nationwide data for Scotland show that IRs of SARS-CoV-2 infections and COVID-19 hospitalisations have consistently been higher in the unvaccinated than in the vaccinated across all relevant age groups during the Delta era. These results, thus, provide a visually powerful, and accessible demonstration of the impact of vaccinations on SARS-CoV-2 infections and COVID-19 hospitalisation throughout the Delta era.
